# Effects of Magnesium Addition on Yield, Quality and Cadmium Accumulation in Pepper Under Cadmium Stress

**DOI:** 10.3390/plants15203109

**Published:** 2026-10-11

**Authors:** Ahua Wang, Fuyuan Ye, Xiaodan Wang, Zijing Wang, Zhaoyan Ren, Guidong Liu, Fengxian Yao, Chen Cheng

**Affiliations:** 1 College of Life Sciences, Gannan Normal University, Ganzhou 341000, China; 18089697156@163.com (A.W.); 18335379768@163.com (Z.R.); liuguidong@gnnu.edu.cn (G.L.); fengxianyao@aliyun.com (F.Y.); 2 Harbin Ecological Environment Monitoring Center of Heilongjiang Province, Harbin 150076, China; 15545133687@163.com; 3 School of Geography and Environmental Engineering, Gannan Normal University, Ganzhou 341000, China; 18470553625@163.com

**Keywords:** cadmium accumulation, cadmium stress, exogenous Mg addition, pepper, yield

## Abstract

To investigate the effects of exogenous magnesium (Mg) addition on cadmium (Cd) accumulation, yield, and quality of pepper under Cd stress, two pepper cultivars, Hongtianhu 101 and Yuefeng 750, which differ greatly in their Cd accumulation capacities, were used as experimental materials. Based on Hoagland nutrient solution, three treatments were established: Cd treatment, Cd + Mg treatment, and a control treatment without Cd or Mg addition (CK). A seedling-stage hydroponic experiment and a pot experiment from the seedling stage to the mature stage were conducted using Hoagland nutrient solutions with different formulations. Indicators including dry matter production, yield, fruit quality, and Cd content in different organs of pepper were systematically determined. The results showed that, compared with the CK treatment, Cd treatment significantly decreased the photosynthetic pigment content, total dry matter weight, and yield at the mature stage, while significantly increasing leaf oxidative stress, Cd accumulation in different organs, and total Cd accumulation, accompanied by reduced fruit quality. However, Mg addition under Cd stress (Cd + Mg treatment) effectively increased the photosynthetic pigment contents and total dry matter weight at the mature stage, resulting in a significant increase in yield of 49.7–54.9%. Meanwhile, Mg addition reduced Cd accumulation in all plant organs, thereby significantly decreasing the total Cd accumulation by 17.2%–58.0%, markedly alleviating the degree of leaf oxidative stress, and improving pepper quality. Nevertheless, none of the above indicators fully recovered to the levels observed in the CK treatment. In summary, Mg addition under Cd stress alleviated the adverse effects of Cd stress in pepper plants, as indicated by improved growth performance, reduced Cd accumulation, and enhanced fruit quality. This study provides a theoretical basis for the production of high-yield and high-quality peppers in Cd-contaminated areas.

## 1. Introduction

Cadmium (Cd) is one of the most toxic non-essential trace elements to humans. Owing to its high hydrophilicity and mobility, Cd in agricultural soils is readily taken up by crop roots, translocated to above-ground tissues, and enriched in edible organs [1]. Thus, Cd stress not only significantly inhibits crop growth and development, leading to reduced yield, but also leads to Cd accumulation in crops, which then enters the human body through the food chain. Dietary exposure to Cd poses a serious threat to human health and has been confirmed as an important risk factor for multi-organ functional damage [2].

According to the National Soil Pollution Survey Bulletin released by the Ministry of Environmental Protection and the Ministry of Land and Resources of China in 2014, Cd was the most prevalent inorganic soil pollutant in cultivated land, with an exceedance rate of 7.0% across monitoring sites [3]. Therefore, reducing Cd accumulation in edible crop tissues while maintaining normal plant growth is of great practical significance for agricultural production in Cd-contaminated areas.

Multiple strategies have been proposed to mitigate Cd uptake and Cd-induced phytotoxicity in crops. Soil washing technology can remove Cd from contaminated soils; however, it is associated with high costs and potential risks of soil structure deterioration [4]. Alternative agronomic approaches include breeding low-Cd-accumulating cultivars, applying biochar, optimizing water and fertilizer management, and applying exogenous mineral nutrients, which may modify Cd speciation and influence plant Cd uptake directly or indirectly [5,6]. Magnesium (Mg), an essential macro-nutrient for plants, has been reported to alleviate Cd toxicity by competing with Cd for root absorption sites, enhancing antioxidant defense systems, and promoting chlorophyll synthesis [7,8]. Therefore, Mg shows promising application potential for reducing Cd accumulation in crops grown under Cd stress.

Pepper is an extensively cultivated vegetable in China, with a planting area exceeding 2.10 million hm^2^, and it exhibits a relatively high Cd accumulation capacity among solanaceous vegetables [9,10]. The alleviative effects of exogenous Mg addition under Cd stress have been documented in rice [11], wheat [12], and pak choi [13]. However, systematic research regarding the effects of Mg addition on Cd stress mitigation in pepper remains limited.

In this study, two pepper cultivars with distinct Cd accumulation characteristics were used to investigate the effects of Mg addition under Cd stress on Cd uptake and translocation, plant growth, and fruit quality. This study aims to provide theoretical support for improving pepper production and reducing Cd accumulation in edible tissues under Cd stress conditions.

## 2. Results

### 2.1. Yield

Compared with CK, Cd treatment significantly decreased pepper yield by 52.4–56.5%. Relative to the Cd treatment, Cd + Mg treatment significantly increased yield by 49.7–54.9%, although yield remained significantly lower than that of CK (*p* < 0.05, Table 1). Regarding yield components, Cd stress significantly reduced fruit number per plant by 32.9–43.4% and single-fruit weight by 16.0–36.1% compared with CK. Under Cd stress, Mg addition increased fruit number by 34.9–39.2% and single-fruit weight by 11.7–11.9%.

### 2.2. Fruit Quality

Compared with CK, Cd treatment decreased fruit soluble sugar, titratable acidity, vitamin C, and soluble protein contents by 6.2–30.9%, 46.8–51.7%, 65.1–80.0% and 39.9–61.3%, respectively (Table 2). Under Cd stress, Mg addition increased these quality indicators by 10.3–35.6%, 49.6–55.0%, 24.0–117.7%, and 38.5–60.0%, respectively, and the magnitude of such improvements was greater in Hongtianhu 101 than in Yuefeng 750.

### 2.3. Dry-Matter Production

Relative to CK, Cd treatment significantly reduced total dry-matter weight by 18.8–41.0% at the seedling stage and by 30.2–44.1% at the mature stage (Table 3). Under Cd stress, Mg addition increased total dry-matter weight by 11.0–32.8% (seedling stage) and 19.1–27.5% (mature stage). Cd stress significantly inhibited dry-matter accumulation in roots, stems and leaves at both developmental stages (except roots and stems of mature Yuefeng 750). Mg addition improved dry-matter accumulation in individual organs under Cd stress, yet these parameters did not recover to CK levels.

### 2.4. Cd Concentration in Different Organs

**Figure 1:**
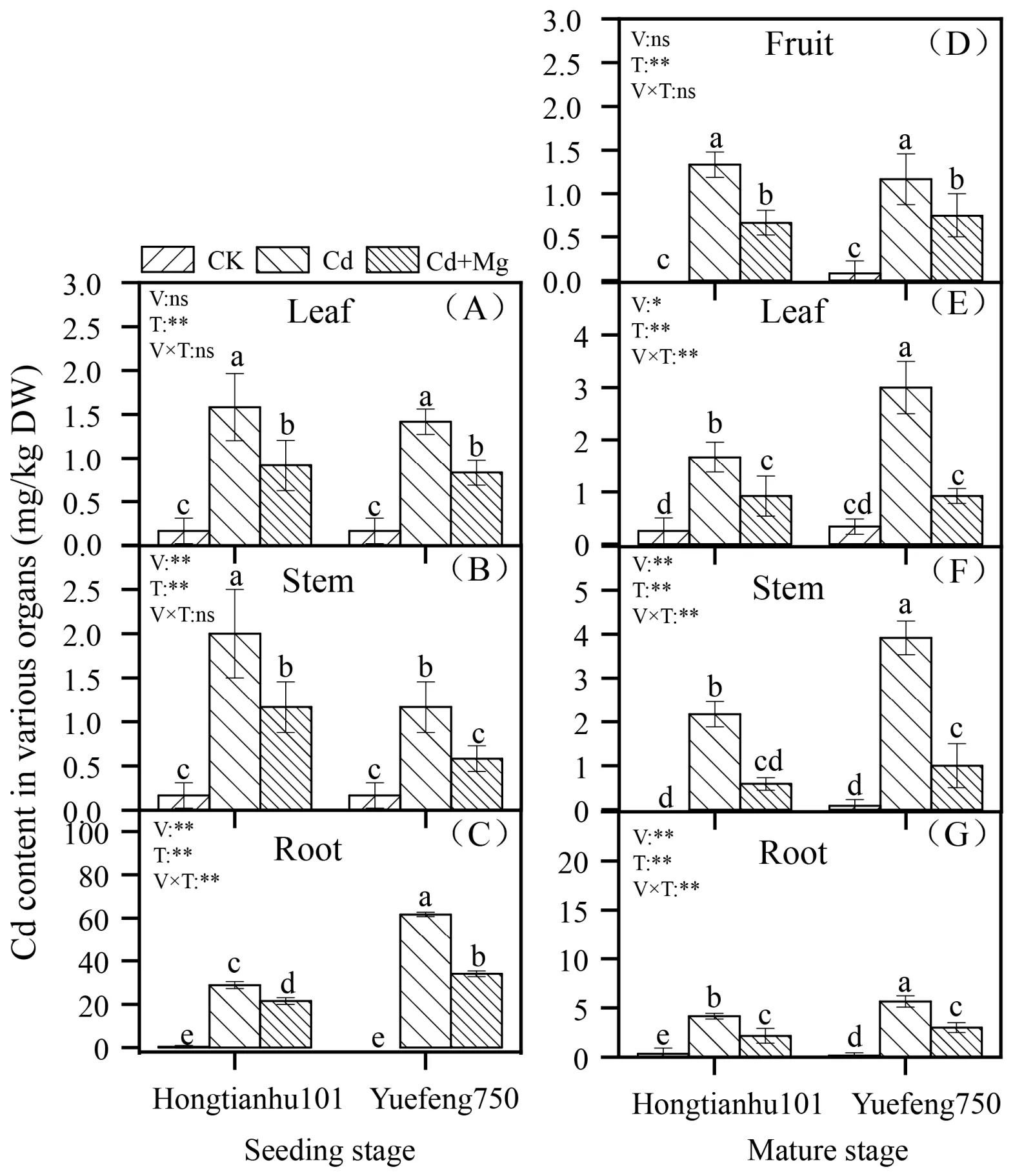
Effects of Mg addition on Cd content in various organs of pepper under Cd stress. Data are presented as mean ± SD (*n* = 3). Different lowercase letters indicate significant differences among treatments (*p* < 0.05). CK, control; Cd, Cd stress; Cd + Mg, Cd stress with Mg addition. Panels (**A**–**C**) represent Cd content in leaf, stem, and root of pepper at the seedling stage, while panels (**D**–**G**) represent Cd content in fruit, leaf, stem, and root of pepper at the mature stage. Significant main and interactive effects are denoted by * (*p* < 0.05) and ** (*p* < 0.01), respectively; ns indicates non-significant difference.

Cd treatment significantly increased Cd concentrations in roots, stems, leaves and fruits at the seedling and mature stages compared with CK (Figure 1). Under Cd stress, Mg addition significantly reduced Cd concentrations in all organs at both stages. Across Cd-containing treatments organ Cd concentrations followed the order root > stem > leaf > fruit. Genotypic differences in Cd accumulation were evident: compared with Hongtianhu 101, Yuefeng 750 exhibited 176.7–200%, 214.3–300%, 62.5–71.4%, and 112.5–114.3% higher Cd concentrations in roots, stems, leaves and fruits at maturity, respectively (excluding CK).

### 2.5. Total Plant Cd Accumulation

**Figure 2:**
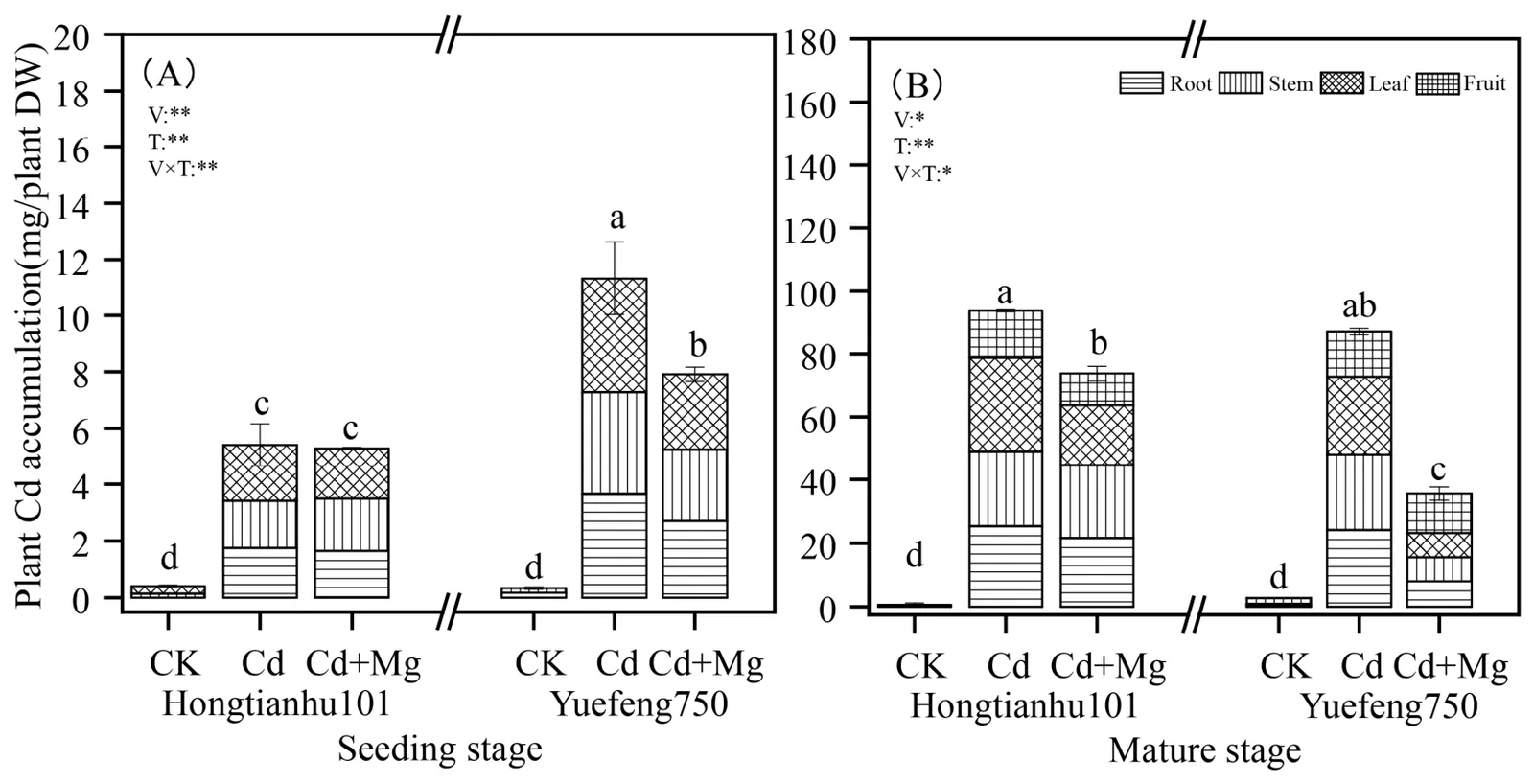
Effects of Mg addition on plant Cd accumulation of pepper under Cd stress. Bars represent mean ± SD (*n* = 3). Different lowercase letters indicate significant differences among treatments (*p* < 0.05). Panels (**A**,**B**) represent Cd accumulation in pepper plants at the seedling stage and mature stage, respectively. Significant main and interactive effects are denoted by * (*p* < 0.05) and ** (*p* < 0.01), respectively; ns indicates non-significant difference.

Compared with CK, Cd treatment significantly increased the total Cd accumulation in pepper plants at the seedling and mature stages (Figure 2). Under Cd stress, exogenous Mg application significantly decreased the total Cd accumulation in mature pepper plants by 17.2%–58.0% in comparison with the single Cd treatment. For the organ-specific distribution of Cd, leaves were the dominant Cd-accumulating organ, contributing 31.2%–37.8% to the total plant Cd accumulation in mature plants across all treatments except CK, followed by stems and roots. By contrast, fruits showed the lowest Cd accumulation proportion, only accounting for 13.0%–18.9%. Moreover, cultivar difference analysis indicated that Yuefeng 750 exhibited distinctly higher total Cd accumulation than Hongtianhu 101 at the seedling stage.

### 2.6. Photosynthetic Pigment Contents

Cd stress decreased contents of chlorophyll a, chlorophyll b, and carotenoid contents at the seedling and mature stages, with significant reductions observed at maturity (Table 4). Mg addition under Cd stress increased photosynthetic pigment contents; chlorophyll b and carotenoid contents were significantly higher in Cd + Mg-treated plants than in Cd-treated plants at maturity, although they remained below CK levels.

### 2.7. Physiological Responses to Stress

#### 2.7.1. Malondialdehyde (MDA) and Proline (Pro) Contents

**Figure 3:**
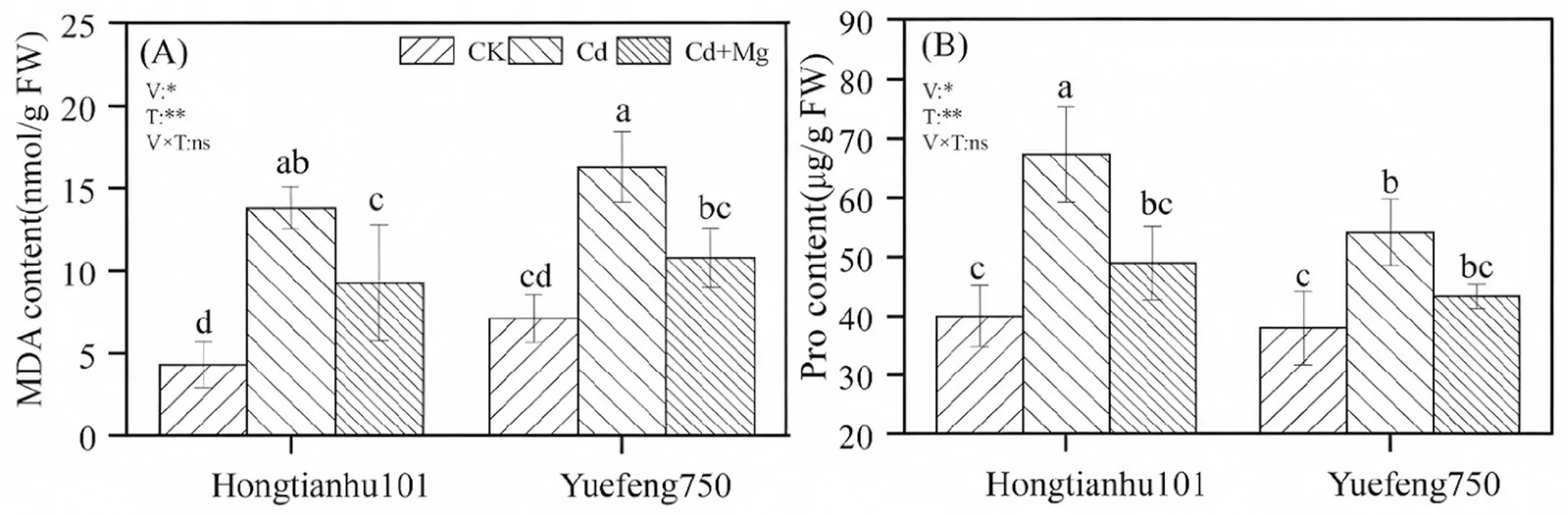
Effects of Mg addition on leaf MDA and Pro content in pepper seedling stage under Cd stress. Different letters above the bars indicate significant differences (*p* < 0.05). Panels (**A**,**B**) show MDA content and Pro content in pepper leaves at the seedling stage, respectively. Significant main and interactive effects are denoted by * (*p* < 0.05) and ** (*p* < 0.01), respectively; ns indicates non-significant difference.

Compared with CK, Cd stress significantly increased seedling-leaf MDA and Pro contents by 129.4–220.4% and 42.3–68.1%, respectively. Under Cd stress, Mg addition reduced MDA and Pro contents by 32.9–33.7% and 19.8–27.3%, respectively, compared with Cd treatment, although both remained higher than CK (Figure 3).

#### 2.7.2. Antioxidant Enzyme Activities

**Figure 4:**
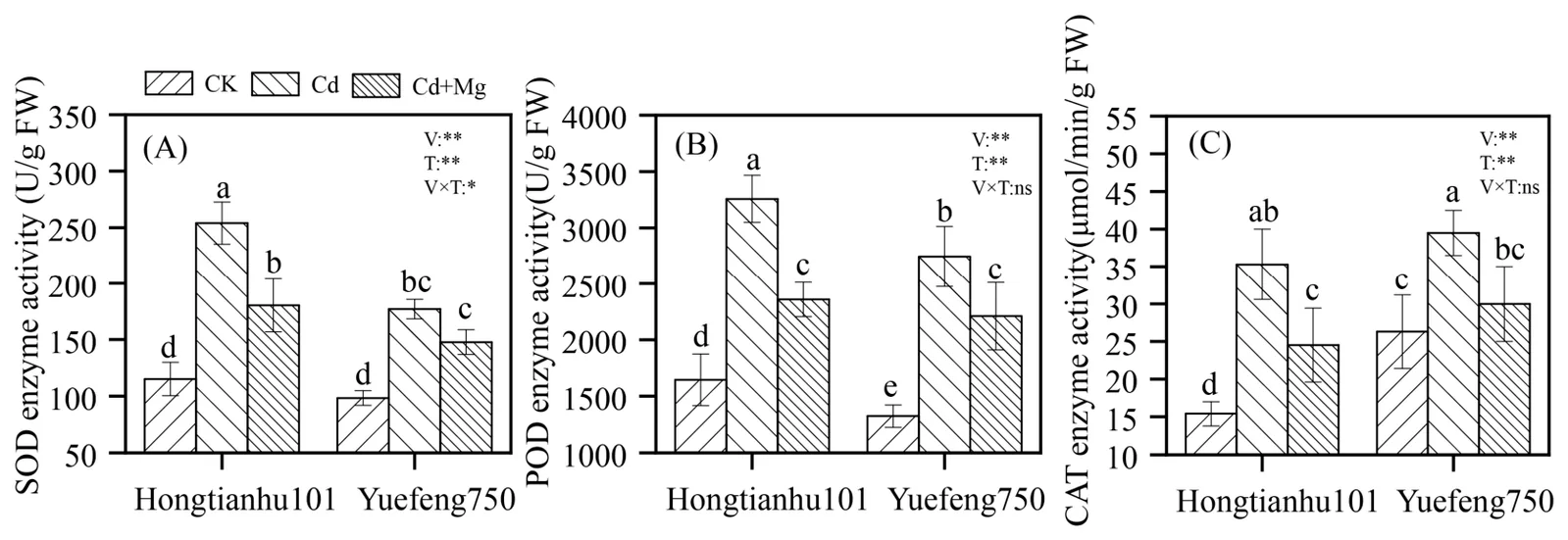
Effects of Mg addition on leaf antioxidant enzyme (SOD, POD, CAT) activity in pepper seedling stage under Cd stress. *p* < 0.05, * *p* < 0.01, ** *p* < 0.001. Different lowercase letters indicate significant differences among treatments (*p* < 0.05). Panels (**A**–**C**) represent SOD, POD and CAT enzyme activity in pepper leaves at the seedling stage, respectively. Significant main and interactive effects are denoted by * (*p* < 0.05) and ** (*p* < 0.01), respectively; ns indicates non-significant difference.

Cd exposure significantly increased superoxide dismutase (SOD), peroxidase (POD), and catalase (CAT) activities in seedling leaves compared with CK. Mg addition under Cd stress significantly reduced these elevated enzyme activities by 16.5–29.0% (SOD), 19.5–27.6% (POD), and 24.0–30.6% (CAT), respectively; however, their activities remained higher than CK (Figure 4).

## 3. Discussion

### 3.1. Impacts of Mg Addition on Pepper Yield Under Cd Stress

Adequate Mg supply promotes dry-matter accumulation in stems, leaves, and fruits and provides a foundation for high crop yield [14]. Previous research has indicated that appropriate Mg nutrition may promote root development, increases fruit number and single-fruit weight, maintains photosynthetic performance, and alleviates yield losses caused by Cd toxicity in pepper [15]. Consistent with this, our study demonstrated that Cd stress drastically suppressed pepper yield, whereas Mg addition under Cd stress reduced this inhibition and increased yield by 49.7–54.9%, mainly through increases in fruit number (34.9–39.2%) and single-fruit weight (11.7–11.9%). Two possible explanations may account for these observations. First, Cd stress induced remarkable accumulation of MDA and Pro and increased antioxidant enzyme activities in seedling leaves; whereas Mg addition decreased leaf MDA content by 32.9–33.7%, suggesting improved plant stress tolerance (Figure 3 and Figure 4). Second, Cd stress reduced photosynthetic pigment contents and dry-matter accumulation, while Mg addition increased photosynthetic pigment levels and dry-matter production (Table 3 and Table 4). Therefore, Mg addition may alleviate Cd-induced growth suppression by improving root performance and antioxidant defense capacity, thereby increasing pepper yield [16]. Nevertheless, growth-related traits under Cd + Mg were still inferior to CK, indicating that sole Mg addition alone cannot fully eliminate persistent Cd-induced damage in pepper under the experimental conditions [17].

### 3.2. Impacts of Mg Addition on Pepper Fruit Quality Under Cd Stress

As an essential mineral nutrient, Mg participates in carbohydrate metabolism and enzyme activation and plays an important role in fruit quality formation [18]. Our results revealed that Cd stress significantly reduced soluble sugar, titratable acidity, vitamin C, and soluble protein contents in pepper fruits, with cultivar-dependent variation; Mg addition partially restored these quality parameters (Table 2). These observations are consistent with previous findings [19,20]. First, Mg may serve as a cofactor for key metabolic enzymes and may regulate sugar, organic-acid, and vitamin C metabolism, facilitating the accumulation of sugars, organic acids, and vitamin C in fruits under Cd stress [21]. Second, Mg may contribute to maintaining ribosome structure and function, supporting protein synthesis and alleviating Cd-induced impairment of nutrient metabolism in fruits [22]. Collectively, Mg addition may improve pepper fruit quality by regulating metabolic processes under Cd stress.

### 3.3. Impacts of Mg Addition on Cd Accumulation in Pepper

Previous research has reported that Mg may mitigate Cd phytotoxicity and promote pepper growth by reducing Cd uptake and translocation to aboveground and edible tissues [23]. In the present study, we directly demonstrated that Cd treatment increased total plant Cd accumulation, whereas Mg addition reduced total Cd accumulation in mature pepper plants by 17.2–58.0%, which is consistent with previous reports [24,25]. Two plausible mechanisms have been proposed in previous studies. First, as a divalent cation, Mg may compete with Cd for root absorption sites and transmembrane transport systems, potentially suppressing Cd uptake by plant roots [26]. Second, Mg has been reported to enhance phytochelatin synthesis, maintain cell membrane integrity, and upregulate genes involved in vacuolar sequestration, which may contribute to Cd chelation and immobilization in root cell walls and vacuoles and limit Cd translocation from roots to shoots [27]. In the present study, Cd predominantly accumulated in pepper roots, followed by stems and leaves, with the lowest proportion detected in fruits. It has been hypothesized that Mg may enhance tonoplast H^+^-ATPase activity and regulate the expression of HMA3 homologs, thereby promoting vacuolar Cd sequestration in roots and potentially limiting Cd translocation to aerial organs via the xylem. These mechanisms may partially explain the observed organ-specific Cd distribution pattern (root > stem > leaf > fruit) [28]. However, these mechanisms involving phytochelatin synthesis, vacuolar sequestration, tonoplast H^+^-ATPase activity, and HMA3 homologs were not directly measured in this study and should therefore be considered hypotheses based on previous literature rather than direct evidence from the present work. Measurement of Mg concentrations in roots, shoots, and fruits would further strengthen the physiological interpretation; however, these data were not available in the present study and their measurement should be prioritized in future work. In this study, Mg addition reduced Cd accumulation in edible tissues and improved pepper yield and quality under the artificial substrate conditions. However, validation under naturally Cd-contaminated field soils is still required.

While the present study provides direct physiological evidence for the effects of Mg addition under Cd stress, several limitations should be acknowledged when interpreting these findings. First, the absence of a Mg-only control means that the independent effects of Mg in the absence of Cd could not be fully separated from its effects under Cd exposure. Therefore, the conclusions of this study are limited to Cd-stressed conditions. Second, because soluble protein content was not determined, enzyme activities were expressed on a fresh weight basis. Although this approach is widely used in plant stress physiology [29,30], variations in extractable protein among samples may influence the interpretation of enzyme activities [31]. Therefore, future studies should include Mg-only treatments and protein-normalized enzyme activity measurements to further clarify the physiological mechanisms underlying Mg responses.

## 4. Materials and Methods

### 4.1. Plant Materials and Reagents

Two pepper cultivars were selected for this study: the Cd-low-accumulating cultivar Hongtianhu 101 (Seminis Seeds (Beijing) Co., Ltd., Beijing, China) and the Cd-high-accumulating cultivar Yuefeng 750 (Jiangxi Yuefeng Seed Industry Co., Ltd., Nanchang, China). The Cd source, CdSO_4_·8/3H_2_O (purity 99%), was purchased from Shanghai Aladdin Biochemical Technology Co., Ltd. (Shanghai, China), and the Mg source, MgSO_4_·7H_2_O, was obtained from Shanghai Macklin Biochemical Co., Ltd. (Shanghai, China).

### 4.2. Experimental Design

The experiment was conducted using a Hoagland nutrient solution system, and three treatments were established: (1) Control (CK): full-strength Hoagland nutrient solution without added Cd or Mg. Mg^2+^ was supplied only by the basal nutrient solution, with a total concentration of 2 mmol L^−1^. (2) Cd treatment: full-strength Hoagland nutrient solution supplemented with 3 μmol L^−1^ Cd^2+^ (added as CdSO_4_) without Mg addition, with a total Mg^2+^ concentration of 2 mmol L^−1^. (3) Cd + Mg treatment: full-strength Hoagland nutrient solution supplemented with 3 μmol L^−1^ Cd^2+^ (added as CdSO_4_) and 8 mmol L^−1^ Mg^2+^ (added as MgSO_4_), resulting in a total Mg^2+^ concentration of 10 mmol L^−1^ (2 mmol L^−1^ basal + 8 mmol L^−1^ supplemental). The full-strength Hoagland nutrient solution consisted of working solution A (4 mmol L^−1^ Ca(NO_3_)_2_·4H_2_O and 6 mmol L^−1^ KNO_3_), working solution B (2 mmol L^−1^ MgSO_4_·7H_2_O and 1 mmol L^−1^ NH_4_H_2_PO_4_), and working solution C (80 μmol L^−1^ NaFe-EDTA, 46.3 μmol L^−1^ H_3_BO_3_, 9.5 μmol L^−1^ MnSO_4_·H_2_O, 0.8 μmol L^−1^ ZnSO_4_·7H_2_O, 0.3 μmol L^−1^ CuSO_4_, and 0.02 μmol L^−1^ (NH_4_)_6_Mo_7_O_24_). The pH of all nutrient solutions was adjusted to 6.2 ± 0.1 using 1.0 mmol L^−1^ NaOH or HCl.

Pepper seeds were disinfected in 0.2% KMnO_4_ solution for 20 min on 8 July 2023, rinsed thoroughly with distilled water, soaked in tap water for 24 h, and rinsed again. The seeds were sown in 72-cell plug trays and grown in a plant growth chamber under 16 h a light/8 h dark photoperiod with day/night temperatures of 26 °C/20 °C.

Seedling-stage hydroponic experiment: Twenty days after sowing (29 July), healthy and uniformly growing seedlings with 1–2 true leaves were randomly selected and transferred to a hydroponic system in a plant growth chamber. The hydroponic containers were black plastic containers (length × width × height = 35 cm × 27 cm × 13 cm) filled with 5 L of half-strength Hoagland nutrient solution. Seedlings were fixed in custom-made rectangular white sponge plates containing 16 holes arranged in 4 rows and 4 columns, with one seedling per hole. The sponge plates tightly covered the openings of the black plastic containers, which were identical in size. Each black plastic container was considered an independent experimental unit. Each treatment included three independent hydroponic containers, representing three biological replicates (*n* = 3). The 16 seedlings within the same container shared the same nutrient solution and were therefore not considered independent replicates. Four days after transplantation, the nutrient solution was replaced with the full-strength formula, and the corresponding Cd or Cd + Mg treatments were added to the nutrient solution. Treatments were applied at the container level, and all seedlings within the same container received the same treatment. The nutrient solution was renewed every 2 days, and the hydroponic experiment continued until 27 August.

Seedling-to-maturity pot experiment: On 3 September, seedlings subjected to different treatments during the seedling-stage hydroponic experiment were transplanted into a pot system. The pots were resin gallon pots (bottom diameter: 18.5 cm; top diameter and height: 23.5 cm) filled with Danish Pindstrup substrate to approximately 80% capacity. One plant was grown per pot, and each treatment included 10 independent pots, representing 10 biological replicates (*n* = 10). Each pot was considered an independent experimental unit, and individual plants within pots were not treated as multiple independent replicates. Full-strength Hoagland nutrient solution was introduced every 2 days. Six days after transplantation, the corresponding Cd or Cd + Mg treatments were supplied through irrigation with nutrient solution, consistent with the seedling-stage treatments. Treatments were applied at the pot level. Each nutrient solution application continued until the solution drained thoroughly from the bottom of the pot to maintain relatively stable element concentrations in the substrate. The experiment continued until 1 January 2024.

Sampling and replication: At the seedling stage (end of the hydroponic experiment), the hydroponic container was considered the independent replicate. For each treatment, representative seedlings were randomly selected from each independent container. For destructive measurements, multiple seedlings from the same container were pooled or averaged to obtain one container-level replicate (*n* = 3). At the mature stage (end of the pot experiment), each pot was considered the independent replicate. For each treatment, one representative plant was randomly selected from each independent pot for measurement (*n* = 10). Plants were first rinsed with tap water, and roots were immersed in 20 mmol L^−1^ Na_2_-EDTA solution for 20 min to remove Cd ions adsorbed onto the root surface. Subsequently, plants were washed with deionized water, blotted dry, and used for subsequent measurements. Technical replicates, including repeated instrumental measurements of the same sample or multiple measurements from seedlings within the same container, were averaged before analysis and were not considered independent biological replicates.

The experiment was arranged in a completely randomized design. Both containers and pots were randomly assigned to treatments. To minimize potential positional effects, the positions of containers and pots were rotated once a month during the experimental period. During each rotation, containers and pots were re-randomized, while the assigned treatments remained unchanged.

### 4.3. Measurement of Indicators

#### 4.3.1. Plant Morphology and Photosynthetic Pigment Contents

Plant height and canopy width were measured using a straight ruler (Deli, Ningbo, China). Stem base diameter was determined using a digital vernier caliper (Mitutoyo, Kawasaki, Japan). Fresh leaf samples were extracted with 95% ethanol (Sinopharm Chemical Reagent Co., Ltd., Shanghai, China) under dark conditions. The absorbance of extracts was measured using an ultraviolet-visible spectrophotometer (UV-2600, Shimadzu, Kyoto, Japan) at wavelengths of 665, 649, and 470 nm.

The contents of chlorophyll a, chlorophyll b, and carotenoids were calculated according to the following equations:

Chl a (mg/g FW) = (13.95 × A665 − 6.88 × A649) × V/(1000 × W)

Chl b (mg/g FW) = (24.96 × A649 − 7.32 × A665) × V/(1000 × W)

Car (mg/g FW) = [(1000 × A470 − 2.05 × Chl a − 114.8 × Chl b)/245] × V/(1000 × W)

where A665, A649, and A470 are absorbance values at the corresponding wavelengths, V is the final extraction volume (mL), and W is the fresh weight of leaf samples (g).

#### 4.3.2. Yield and Fruit Quality

Fruits were harvested every 15 days after reaching maturity. The fresh weight of each fruit was recorded, and the cumulative yield per plant was calculated. At the end of the experiment, ten representative mature fruits were randomly sampled from each treatment for quality analysis and assigned to three biological replicates. Fruits within the same biological replicate were pooled and homogenized under ice-bath conditions. Soluble sugar content was determined by anthrone colorimetry [32]. Titratable acidity was determined according to ISO 750:1998. Vitamin C (ascorbic acid) content was determined using the 2,6-dichloroindophenol (DCPIP) titration method. Soluble protein content was assayed using the Coomassie brilliant blue G-250 method [33]. Anthrone, 2,6-dichloroindophenol, and Coomassie brilliant blue G-250 were purchased from Sinopharm Chemical Reagent Co., Ltd. (Shanghai, China).

#### 4.3.3. Dry-Matter Accumulation and Cd Concentration

Plant samples were separated into roots, stems, leaves, and fruits. Samples were first dried at 105 °C for 30 min in an electrothermal blast drying oven (DHG-9070A, Shanghai Yiheng Scientific Instrument Co., Ltd., Shanghai, China), followed by drying at 75 °C to constant weight to obtain organ dry weight. Dried samples were ground with a plant grinder (FW100, Tianjin Taisite Instrument Co., Ltd., Tianjin, China) and passed through a 100-mesh sieve. After digestion, Cd concentration was determined by inductively coupled plasma mass spectrometry (ICP-MS, Agilent 7900, Agilent Technologies, Santa Clara, CA, USA) [34].

#### 4.3.4. Antioxidant Enzyme Activities and Osmotic-Regulating Substances

At the seedling stage, representative leaves from each treatment were randomly selected, rinsed with distilled water, blotted dry, immediately frozen in liquid nitrogen for 1 min, and then stored at −80 °C in an ultra-low-temperature freezer (MDF-U53V, Panasonic, Osaka, Japan). The activities of POD, CAT, and SOD, as well as the contents of MDA and Pro, were determined using commercial assay kits from Suzhou Comin Biotechnology Co., Ltd. (Suzhou, China) through spectrophotometric methods according to the manufacturer’s instructions. The activities of SOD, CAT, and POD were expressed as U/g FW, whereas MDA and Pro contents were expressed as nmol/g FW and μg/g FW, respectively. Each treatment was analyzed using three biological replicates.

### 4.4. Statistical Analysis

All data were presented as mean ± standard deviation. SPSS Statistics 22.0 (IBM, Armonk, NY, USA) was used for statistical analysis, Microsoft Excel 2021 (Microsoft, Redmond, WA, USA) was used for data processing, OriginPro 9.0 (OriginLab, Northampton, MA, USA) was used for plotting. Differences among treatments were evaluated using one-way ANOVA, followed by the LSD multiple comparison test at *p* < 0.05.

## 5. Conclusions

Cd stress decreased pepper yield, increased Cd accumulation in plants, and deteriorated fruit quality compared with the control (CK). Mg addition effectively alleviated these adverse effects by mitigating oxidative injury, increasing photosynthetic pigment levels and dry matter accumulation, and consequently improving yield, decreasing total plant Cd accumulation, and enhancing fruit quality. Under Cd stress, Mg addition also regulated SOD/CAT/POD activities on a fresh weight basis, which may indicate an altered antioxidant response in the tissue. However, none of the measured indicators recovered to the CK level.

It should be emphasized that these findings were obtained under an artificial substrate system with Cd supplied through nutrient solution. This system differs substantially from naturally Cd-contaminated field soils. Therefore, the mitigating effects of Mg addition observed in this study represent preliminary evidence under controlled conditions and cannot be directly extrapolated to naturally Cd-contaminated farmland. Field validation under naturally Cd-contaminated soils is still required before practical recommendations can be proposed.

In summary, Mg addition can alleviate the inhibitory effects of Cd stress on pepper growth under the artificial substrate conditions used in this study. Future studies should explore the combined application of Mg addition with other management strategies in naturally Cd-contaminated soils to verify its practical potential under field conditions.

## Figures and Tables

**Table 1:** Effects of Mg addition on the yield and its constituent factors of Pepper under Cd Stress.

Variety	Treatment	Fruit Number (per Plant)	Single Fruit Weight (g)	Yield (g/Plant)
Hongtianhu101	CK	25.3 ± 3.8 a	8.4 ± 0.8 b	213.2 ± 12.2 b
	Cd	14.3 ± 3.1 c	7.1 ± 0.9 cd	101.4 ± 10.7 d
	Cd + Mg	19.3 ± 3.5 abc	7.9 ± 0.3 bc	151.8 ± 8.5 c
Yuefeng750	CK	25.3 ± 3.1 a	10.1 ± 0.3 a	253.6 ± 23.7 a
	Cd	17.0 ± 3.0 bc	6.5 ± 0.2 d	110.4 ± 16.7 d
	Cd + Mg	23.7 ± 3.2 ab	7.3 ± 0.7 bcd	171.0 ± 16.0 c
Statistical significance				
Variety (V)		ns	ns	*
Treatment (T)		**	**	**
V × T		ns	*	ns

Note: Values in each column are means ± SD (*n* = 3). Means followed by different lowercase letters indicate significant differences among treatments at (*p* < 0.05), determined by two-way ANOVA for each variety. Significant main and interactive effects are denoted by * (*p* < 0.05) and ** (*p* < 0.01), respectively; ns indicates non-significant difference.

**Table 2:** Effects of Mg addition on the quality of pepper under Cd stress.

Variety	Treatment	Soluble Sugar Content (%)	Titratable Acid Content (%)	Vitamin C Content (mg/100 g)	Soluble Protein Content (mg/g)
Hongtianhu101	CK	1.24 ± 0.22 a	3.82 ± 0.48 ab	27.7 ± 6.7 a	0.21 ± 0.05 a
	Cd	0.86 ± 0.05 b	1.84 ± 0.39 b	5.5 ± 0.9 c	0.13 ± 0.01 cd
	Cd + Mg	1.16 ± 0.08 a	2.76 ± 0.24 ab	12.1 ± 1.4 b	0.18 ± 0.01 ab
Yuefeng750	CK	1.34 ± 0.15 a	2.26 ± 0.37 ab	5.4 ± 0.7 c	0.14 ± 0.03 bc
	Cd	1.26 ± 0.11 a	1.20 ± 0.22 ab	1.9 ± 0.1 c	0.05 ± 0.03 e
	Cd + Mg	1.39 ± 0.10 a	1.86 ± 0.23 a	2.3 ± 0.3 c	0.08 ± 0.02 de
Statistical significance					
Variety (V)		**	ns	**	**
Treatment (T)		*	ns	**	**
V × T		ns	ns	**	ns

Note: Values in each column are means ± SD (*n* = 3). Means followed by different lowercase letters indicate significant differences among treatments at (*p* < 0.05), determined by two-way ANOVA for each variety. Significant main and interactive effects are denoted by * (*p* < 0.05) and ** (*p* < 0.01), respectively; ns indicates non-significant difference.

**Table 3:** Effects of Mg addition on dry matter production of pepper under Cd stress.

Variety	Treatment	Seedling Stage				Mature Stage				
		Roots	Stems	Leaves	Sum	Roots	Stems	Leaves	Fruits	Sum
Hongtianhu 101	CK	0.09 ± 0.01 a	0.13 ± 0.01 c	0.46 ± 0.03 ab	0.68 ± 0.04 b	3.1 ± 0.6 a	25.6 ± 1.9 a	23.8 ± 0.8 a	20.8 ± 2.7 b	73.3 ± 5.1 a
	Cd	0.04 ± 0.01 d	0.07 ± 0.01 e	0.29 ± 0.03 e	0.40 ± 0.04 e	2.0 ± 0.2 bc	20.1 ± 2.6 b	18.0 ± 0.9 c	11.1 ± 1.7 d	51.2 ± 2.4 c
	Cd + Mg	0.06 ± 0.01 bc	0.10 ± 0.02 d	0.37 ± 0.02 d	0.53 ± 0.04 d	2.5 ± 0.1 ab	22.8 ± 2.0 b	20.5 ± 1.9 b	15.2 ± 0.8 cd	61.0 ± 4.5 b
Yuefeng 750	CK	0.07 ± 0.01 ab	0.18 ± 0.01 a	0.48 ± 0.03 a	0.74 ± 0.02 a	1.8 ± 0.3 cd	8.8 ± 1.7 c	9.5 ± 1.2 d	26.2 ± 4.1 a	46.2 ± 6.7 c
	Cd	0.05 ± 0.01 cd	0.15 ± 0.01 bc	0.40 ± 0.02 cd	0.60 ± 0.02 c	1.2 ± 0.2 d	6.1 ± 0.5 c	6.6 ± 0.4 e	12.0 ± 1.0 d	25.8 ± 1.1 d
	Cd + Mg	0.06 ± 0.01 bc	0.16 ± 0.01 ab	0.44 ± 0.02 bc	0.67 ± 0.03 b	1.5 ± 0.1 cd	6.5 ± 0.7 c	8.2 ± 0.4 de	16.7 ± 1.3 bc	33.0 ± 2.2 d
Statistical significance										
Variety (V)		ns	**	**	**	**	**	**	*	**
Treatment (T)		**	**	**	**	**	**	**	**	**
V × T		ns	ns	**	*	ns	ns	ns	ns	ns

Note: Values in each column are means ± SD (*n* = 3). Means followed by different lowercase letters indicate significant differences among treatments at (*p* < 0.05), determined by two-way ANOVA for each variety. Significant main and interactive effects are denoted by * (*p* < 0.05) and ** (*p* < 0.01), respectively; ns indicates non-significant difference.

**Table 4:** Effects of Mg addition on photosynthetic pigment content of pepper under Cd stress.

Variety	Treatment	Seedling Stage			Mature Stage		
		Chlorophyll a	Chlorophyll b	Carotenoids	Chlorophyll a	Chlorophyll b	Carotenoids
Hongtianhu101	CK	1.78 ± 0.05 bc	0.65 ± 0.03 bc	0.36 ± 0.01 ab	1.17 ± 0.07 a	0.44 ± 0.03 a	2.20 ± 0.07 a
	Cd	1.57 ± 0.12 d	0.59 ± 0.06 c	0.31 ± 0.01 c	0.99 ± 0.06 bc	0.35 ± 0.04 cd	1.77 ± 0.10 cd
	Cd + Mg	1.66 ± 0.04 cd	0.62 ± 0.02 bc	0.33 ± 0.01 bc	1.10 ± 0.09 ab	0.41 ± 0.02 ab	2.01 ± 0.20 ab
Yuefeng750	CK	2.04 ± 0.08 a	0.75 ± 0.06 a	0.38 ± 0.03 a	1.14 ± 0.04 a	0.38 ± 0.01 bc	2.15 ± 0.12 ab
	Cd	1.88 ± 0.09 ab	0.65 ± 0.01 b	0.33 ± 0.02 bc	0.90 ± 0.05 c	0.32 ± 0.03 d	1.63 ± 0.09 d
	Cd + Mg	1.95 ± 0.10 ab	0.72 ± 0.01 a	0.37 ± 0.02 a	1.07 ± 0.04 ab	0.37 ± 0.02 bc	1.95 ± 0.08 bc
Statistical significance							
Variety (V)		**	**	**	ns	**	ns
Treatment (T)		*	**	**	**	**	**
V × T		ns	ns	ns	ns	ns	ns

Note: Values in each column are means ± SD (*n* = 3). Means followed by different lowercase letters indicate significant differences among treatments at (*p* < 0.05), determined by two-way ANOVA for each variety. Significant main and interactive effects are denoted by * (*p* < 0.05) and ** (*p* < 0.01), respectively; ns indicates non-significant difference.

## Data Availability

The original contributions presented in this study are included in the article. Further inquiries can be directed to the corresponding author.
